# Personal Views of Aging Among Informal Caregivers of People with Dementia and Non-Caregivers: Gauging the Role of Individual Characteristics and Caregiving-Related Burden

**DOI:** 10.3390/healthcare13222884

**Published:** 2025-11-12

**Authors:** Elena Carbone, Serena Sabatini, Federica Piras, Enrico Sella, Beth Fairfield, Salvatore Bazzano, Flavio Busonera, Lucia Borgia, Linda Clare, Erika Borella

**Affiliations:** 1 Department of General Psychology, University of Padova, 35131 Padova, Italy; enrico.sella@unipd.it (E.S.); erika.borella@unipd.it (E.B.); 2 Faculty of Health and Medical Sciences, School of Psychology, University of Surrey, Guildford GU2 7XH, UK; s.sabatini@surrey.ac.uk; 3 Neuropsychiatry Laboratory, Clinical Neuroscience and Neurorehabilitation Department, IRCCS Santa Lucia Foundation, 00179 Rome, Italy; pirasfphd@gmail.com; 4 Department of Humanities, Federico II University of Naples, 80138 Naples, Italy; beth.fairfield@unina.it; 5 Geriatric Unit, Medical Department, Dolo Hospital, ULSS3 Serenissima, 30031 Dolo, Italy; salvatore.bazzano@aulss3.veneto.it (S.B.); flavio.busonera@aulss3.veneto.it (F.B.); 6 Associazione Malattia di Alzheimer Padova Onlus, 35135 Padova, Italy; centroalzheimer@casamadreteresa.it; 7 Department of Health and Community Sciences, University of Exeter Medical School, Exeter EX4 4PY, UK; l.clare@exeter.ac.uk

**Keywords:** felt age, awareness of age-related change, dementia caregivers, caregiving strain, individual differences

## Abstract

**Background:** Caring for dependent older relatives is thought to influence caregivers’ personal views of aging (VoA)—that is, perceptions regarding their own aging self. This study aimed to examine personal VoA, particularly felt age (FA) and awareness of age-related change (AARC), in caregivers of people with dementia (PwD) compared to non-caregivers, and to ascertain their relationship with caregiving-related burden and distress among dementia caregivers. **Methods:** Seventy dementia caregivers and 94 non-caregivers (age range: 45–85 years) reported their FA and completed a questionnaire assessing awareness of age-related gains (AARC-Gains) and losses (AARC-Losses) and a mood measure. Dementia caregivers’ burden and distress were also relieved. **Results:** No differences emerged between dementia caregivers and non-caregivers’ personal VoA. Different sociodemographic and health-related factors were related to AARC-Gains or AARC-Losses, but not felt age, in each group. AARC-Gains were associated with social status among non-caregivers, whereas AARC-Losses were related to chronological age and subclinical depression in non-caregivers, and to social status, self-rated health, and burden in dementia caregivers. A path model revealed a direct effect of burden, social status, and self-rated health, as well as an indirect one of subclinical depression through burden, on caregivers’ AARC-Losses. **Conclusions:** These findings confirm the interplay between VoA, sociodemographic, and health-related factors in adulthood and older age. They, then, suggest that the strains derived from caring for a PwD influence dementia caregivers’ personal VoA, particularly when their awareness of age-related losses is concerned.

## 1. Introduction

Informal caregiving refers to the provision of unpaid care for relatives with chronic illness, physical disabilities, or psychological conditions who cannot care for themselves and represents a critical, protracted, and stressful situation impacting the quality of life. Indeed, caregivers often experience a multidimensional negative response to the physical, psychological, emotional, and social stressors associated with caregiving, called caregiving burden [1]. The most recent global rates have shown that the median prevalence of burden among informal caregivers is 49.26%, and the median prevalence of related disease (i.e., depression) outcomes is 33.35% [2]. Such findings underline the importance of acknowledging mental health and factors that can contribute to burden in this population as key priorities for healthcare systems worldwide.

The aging population and the consequent rise in neurocognitive diseases, such as dementia, among older adults represent a growing social emergency. Due to its neurodegenerative nature, and progressive impact on a range of abilities necessary for performing daily living skills, dementia is one of the major causes of disability and dependency among older adults.

To date, the high level of care needed by community-dwelling people with dementia (PwD) is predominantly provided by informal caregivers [3]. Dementia caregivers are not only required to assist relatives with dementia-related progressive daily needs (e.g., helping with everyday basic and instrumental activities, monitoring safety), but also manage the cognitive, behavioral, and psychological symptoms of such a disorder, which represent one of their major sources of distress [4,5,6,7,8]. Dementia caregivers are thus known to report poorer psychological outcomes (e.g., depression, low psychological well-being) and greater physical impairments—with increasing risk for their health as the neurocognitive disease of the PwD progresses—compared to both informal caregivers taking care of individuals with physical illnesses (e.g., cancer, stroke) or psychiatric disorders (e.g., schizophrenia) [6,7,8,9] and non-caregiving peers [9,10,11]. As the number of PwD is expected to reach about 152 billion cases by 2050 worldwide, and thus the number of informal caregivers to increase, addressing caregiving-related sequelae among dementia caregivers is crucial [4].

According to a recently proposed theoretical framework [12] and previous findings [13,14,15,16], being in contact with and providing care for older relatives could be among the interpersonal/family experiences that may shape an individuals’ representations, experiences, and expectations regarding their own aging self, namely personal views of aging (VoA [12]).

Personal VoA, typically operationalized in terms of attitudes towards one’s own aging, i.e., individuals’ evaluations of their own aging process [17], or felt age, i.e., the discrepancy between an individual’s felt age (how old someone feels) and actual chronological age [17], has been shown to strongly impact mental and physical outcomes [18] as well as resilience to stressors [19] in adulthood and older age. Because of this evidence and the negative associations between caregiving burden and mental and physical health, increasing attention has thus been devoted to examining correlates of personal VoA among adults/older adults taking care of a sick, dependent older parent. For instance, informal caregivers’ attitudes towards their own aging related to physical change and psychosocial loss were found to be explained by high depressive symptoms and caregiver burden, but a high burden and a younger chronological age were found to be associated with more positive attitudes towards their own aging related to psychological growth [20]. Informal caregivers’ negative attitudes towards older adults were predicted, alongside older chronological age and poorer health, also by higher levels of perceived distress [21], whereas caregivers’ positive attitudes towards their own aging were shown to be associated, among others, with self-reported resilience and social support [22].

Only a few studies focused on informal caregivers of PwD to ascertain the interplay between caregiving-related outcomes and personal VoA, however. For example, Kim and Bolkan [23] showed that dementia caregivers’ positive attitudes toward their own aging were associated with better emotional health (lower negative emotions and higher positive emotions). Sabatini et al. [24], instead, found an older felt age (i.e., feeling older than one’s own chronological age) among dementia caregivers related to higher depressive symptoms, health conditions and generalized distress.

This handful of studies suggests nuanced associations between the strains of caring for an older dependent relative and informal caregivers’ VoA, which might point in both negative [21,24] and positive directions [20,23]. However, dementia caregivers’ personal VoA were assessed mainly in terms of attitudes towards one’s own aging or felt age so far, which returns a simplistic (one-dimensional) evaluation of personal VoA. Using more comprehensive measures of personal VoA may help to clarify the picture and are worth considering.

Moreover, little is known regarding individual differences in personal VoA between informal caregivers and peers who have never taken this role. The few studies that examined this issue [20,25,26] found no substantial differences between dementia caregivers’ and non-caregiving peers’ personal experiences of aging.

Therefore, understanding whether caring for a PwD impacts caregivers’ personal VoA as compared to non-caregiving warrants investigation. Considering sociodemographic and health factors known to relate to VoA [17,27] would also permit delineation of the potential differences between these groups, and the extent to which different VoA measures might relate to the distress and burden experienced by dementia caregivers is also worth investigating.

One aim of the present study was thus to examine whether informal caregivers of PwD and non-caregiver peers differ in their personal VoA. Here, alongside the widely used concept of felt age, we included the more recent concept of the awareness of age-related change (AARC) construct among personal VoA [12]. AARC is a multidimensional construct that expresses “a person’s state of awareness that their behavior, level of performance, or way of experiencing life have changed as a consequence of having grown older” [28]. It is measured with a homonymous questionnaire [29], translated into several languages and showing good psychometric properties and cross-cultural consistency [30]. It captures both positive (AARC-Gains) and negative (AARC-Losses) subjective evaluations of one’s own aging across different domains of functioning (cognitive, physical, socioemotional), and thus might represent a more comprehensive, multidimensional evaluation of individuals’ perceptions of their own aging self.

On the one hand, we could expect dementia caregivers to feel older and experience lower AARC-Gains and higher AARC-Losses than non-caregivers. Being confronted not only with the striking, multifaceted deterioration nature of dementia disease but also with the specific physical and psychological stressors associated with caring for a PwD and the social stigma accompanying dementia [31], might lead dementia caregivers to experience more negative perceptions and expectations regarding their own aging [21,24]. On the other hand, experiences and confrontation with the deterioration of the relative with dementia they are taking care of might lead dementia caregivers to give value to their resources and current status, thereby experiencing more positive perceptions and expectations regarding their own aging self [16,25,32]. We could thus also expect dementia caregivers to report higher AARC-Gains than non-caregiving peers. However, as previous studies demonstrated [20,25,26], small or negligible effects of caregiving, compared with non-caregiving, on awareness of age-related changes and felt age could also be expected.

Another aim was to explore whether classical individual characteristics assumed to relate to VoA [16,17,20,21,27], such as sociodemographic (age, education, and occupation) and health factors, could differently influence felt age, AARC-Gains, and AARC-Losses among caregivers and non-caregiving peers. Alongside poorer health outcomes in dementia caregivers compared with non-caregivers [9,10,11], we could also expect an older age, a poorer social status, and worse health outcomes to be related to more negative personal VoA [18,27], especially among dementia caregivers [16,20,21,33].

Finally, we examined whether and to what extent dementia caregivers’ personal VoA were associated with their caregiving-related strains in terms of both caregiver burden -as measured by the Caregiver Burden Inventory [34], and the distress experienced -as measured by the Neuropsychiatric Inventory Distress scale [35,36]. In line with previous studies, although using one-dimensional measures of personal VoA [20,21,24] rather than multidimensional ones used here, we could expect higher burden and distress experienced by dementia caregivers associated with feeling older and experiencing lower AARC-Gains and higher AARC-Losses. We also explored whether and to what extent burden and distress play a role in explaining personal VoA, depending on the VoA facet considered (felt age, AARC-Gains, and AARC-Losses). In line with literature [20,21,24], we expect both burden and distress to impact VoA. At the same time, burden, as measured here, captures broader social, physical, and psychological constraints and consequences of caring for a PwD, while NPI distress represents more specific challenges related to caring for a PwD, such as managing behavioral and psychological symptoms of dementia [37,38]. It may be thus that burden, more than NPI-distress, contributes to explaining caregivers’ awareness of aging-related changes across different domains of functioning, as assessed by AARC, particularly in terms of greater AARC-Losses.

A path model was also estimated to further explore whether dementia caregivers’ burden and distress likely explain the associations between sociodemographic factors and health indicators and personal VoA.

## 2. Materials and Methods

### 2.1. Participants

Dementia caregivers of community-dwelling PwDs were recruited through collaboration with local dementia care services to include caregivers who were the main care providers for an older person with a dementia diagnosis. During the course of data collection, not only dementia caregivers attending the dementia care center but also additional caregivers seeking support were recruited. Only dementia caregivers providing regular care and assistance (almost daily and more than 9 h weekly [39]) to a relative with dementia were included in the study.

Non-caregivers were recruited through informal networks and word-of-mouth dissemination, ensuring that they had no family members with dementia or had family members with dementia but never had any direct and consistent involvement in their care. During the course of data collection, regular checks were conducted to ensure that the two groups remained as homogeneous as possible in terms of sociodemographic characteristics and socioeconomic status.

Inclusion criteria for both dementia caregivers and non-caregivers included the following: (i) no history of major physical or mental health issues as assessed through a semi-structured interview [40]; (ii) a Montreal Cognitive Assessment-BLIND score ≥ 17 (MoCA-BLIND [41]), indicating no signs of mild/major neurocognitive disorder; (iii) Wechsler vocabulary subtest scores within norms [42]; and (iv) no sign of major depressive symptoms (i.e., a Geriatric Depression Scale score ≤ 5 [43]).

All in all, the study involved 70 informal dementia caregivers (87% females) and 94 non-caregivers (73% females) aged 45 to 85 years.

All participants were Italian native speakers living in central-northern Italy and volunteered for the study, i.e., no compensation was foreseen.

Descriptive statistics of sociodemographic characteristics by group, together with characteristics of dementia caregivers and their care recipients are shown in Table 1.

### 2.2. Materials

#### 2.2.1. Personal Views of Aging

*Awareness of Age-Related Change questionnaire* (AARC; adapted from [29] by Erika Borella, Beth Fairfield, and Serena Sabatini). The present Italian version of the AARC (see also [44]) was translated and adapted from the AARC questionnaire developed by Brothers et al. [29].

Of the total 50 items, 25 assess AARC-Gains and 25 assess AARC-Losses in different life and behavioral domains (i.e., health/physical functioning, cognitive functioning, interpersonal relationships, socio-cognitive and socio-emotional functioning, lifestyle engagement). Participants were asked to rate how much each item applied to them on a 5-point Likert scale (from 1 = not at all to 5 = very much). The dependent variables, AARC-Gains and AARC-Losses scores, were calculated separately by summing the 25 items belonging to the respective subscales (max = 125; Cronbach alpha: AARC-Gains = 0.89, AARC-Losses = 0.89). Higher scores indicate higher AARC-Gains and AARC-Losses, respectively.

*Felt age*. Participants were asked to answer a single-item question: “Please indicate the age that you feel from 0 to 120 years” to provide their felt age. Proportional discrepancy scores (dependent variable) were calculated for each participant using the following formula: felt age—chronological age/chronological age [45]. Negative scores correspond to feeling younger than one’s own chronological age.

#### 2.2.2. Caregiver Burden and Distress

*Caregiver Burden Inventory* [34]. It comprises 24 items divided into 5 dimensions of burden due to caregiving, that is, stress caused by the following: restricting one’s personal time due to caregiving (time-dependence); a sense of failure regarding one’s hopes and expectations compared with peers (developmental); feelings of chronic fatigue and decreased physical health caused by caregiving (physical); conflict of roles concerning one’s job or family (social); and any embarrassment or feeling of shame caused by the relative with dementia (emotional). Each item is rated on a 5-point Likert scale (from 0 = not at all descriptive to 4 = very descriptive). The dependent variable was the sum of the scores for each item (max = 96; Cronbach alpha = 0.88), higher scores indicating a greater caregiver burden.

*Neuropsychiatric Inventory-distress subscale* [35,36,38]. It provides a measure of subjective caregiver distress in relation to 12 behavioral and psychological symptoms characteristic of dementia (i.e., delirium, hallucinations, agitation, dysphoria, anxiety, euphoria, apathy, disinhibition, irritability, motor disturbances, sleep disturbances, food issues), along a 6-point Likert scale (from 0 = none to 5 = severe). We considered the sum of the caregiver’s distress scores for each symptom (max = 60; Cronbach alpha = 0.75) as our dependent variable, with higher scores corresponding to more severe caregiver distress.

#### 2.2.3. Sociodemographic and Health Factors

Chronological age and social status were considered as sociodemographic characteristics. Social status was expressed by the social status index [46], which is indicative of an individual’s class position based on educational attainment, rated on a 7-point Likert scale (1 = elementary school; 7 = graduate/professional training), and occupational status, rated on a 10-point Likert scale (0 = retired; 9 = higher executives, major professionals). As for health status, the GDS scores were used as a measure of subclinical depression. Participants also rated their physical and psychological health by answering the following questions: “How do you rate your overall physical health?” and “How do you rate your overall psychological health?” using a 5-point Likert scale (1 = very poor; 5 = very good). We calculated a composite score expressing overall self-rated health, with higher scores corresponding to better perceived health.

### 2.3. Procedure

After giving written informed consent, all participants attended one individual session lasting about 90 min. The session was conducted remotely (via Zoom or Skype platforms) by a trained experimenter, who guided participants in completing a series of tasks and questionnaires. Participants completed a semi-structured interview that included sociodemographic information, care-recipient and caregiving information (for caregivers only), felt age and physical and psychological health, the MoCA-BLIND, the Wechsler vocabulary subtest, the AARC, and the GDS. Informal caregivers were involved in an additional telephone interview lasting 40 min to complete the NPI and the CBI.

### 2.4. Statistical Analyses

Preliminarily, chi-square and separate analyses of variance (ANOVAs) were run to ascertain differences between dementia caregivers and non-caregivers in terms of gender, chronological age, social status, and health measures (self-rated health, subclinical depression).

To ascertain any differences between caregivers and non-caregivers’ personal VoA, ANOVAs were run with group (caregivers vs. non-caregivers) as the between-subject factor and felt age, AARC-Gains and AARC-Losses scores separately as dependent variables.

Then, to investigate the relationships between sociodemographic (chronological age, social status) and health (self-rated health, subclinical depression) factors, assumed to relate to VoA [16,17,20,21,27,33], linear model (LM) analyses for felt age, AARC-Gains, and AARC-Losses were conducted separately for dementia caregivers and non-caregivers. To identify the more parsimonious model among possible alternatives [47,48], a model comparison approach was used to find the relevant predictors for AARC-Gains and AARC-Losses scores, from a null model (intercept only) to a full model, i.e., including all predictors (see Table 2).

In particular, a null model (m0) was run, followed by a model including chronological age and the social status index (m1), to examine the role of sociodemographics in explaining personal VoA among dementia caregivers and non-caregivers. Subsequently, to assess any additional contribution of health factors to dementia caregivers and non-caregivers’ personal VoA dimensions considered here, the full model (m2) was computed by adding sociodemographic characteristics, self-rated health, and subclinical depression symptoms (see Table 2). For felt age, chronological age was not considered as a predictor since felt age represents the proportional discrepancy between felt age and chronological age, adjusted for chronological age.

To further examine any additional contribution of caregivers’ burden and distress to explaining personal VoA, three additional models were run for dementia caregivers only. As the CBI and the NPI-Distress capture related, yet different sources of caregiving strain [37,38] (i.e., broad personal, social, physical, and emotional constraints and sources of burden related to caregiving or the distress derived specifically from the occurrence of behavioral and psychological symptoms characteristic of dementia, respectively), they were separately and then jointly considered as predictors of personal VoA in the models. Therefore, we again started our analysis with the null model (m0), followed by the model with sociodemographic characteristics (m1) and then the model (m2) also including health measures (see Table 2). Subsequently, we ran three additional models as follows: one adding only CBI scores (m3), another adding only NPI-Distress scores (m4), and a full model (m5), including both CBI and NPI-Distress scores (see Table 2). Here again, for felt age, chronological age was not considered in the models.

All LMs were run using the lm() function in R software version 4.3.1 [49]. Each model was compared to the previous one using the Akaike Information Criterion (AIC [48,50]). The most plausible model for each variable of interest was considered as the one with the lowest AIC value and the larger AIC weight (MuMIn package version 1.47.5 [48]). To account for issues regarding multiple testing, the alpha level was set to *p* < 0.017.

Finally, depending on the results of the model comparison, we exploratorily ran path model testing the indirect effects of self-rated health and subclinical depression through burden and/or distress, on personal VoA (felt age, AARC-Gains, and/or AARC-Losses) among dementia caregivers. The “lavaan” package [51] and maximum likelihood were used to estimate the parameters of the model.

## 3. Results

Results from preliminary analyses showed that the two groups did not differ in terms of gender distribution, χ^2^_(1)_ = 3.81, *p* = 0.051, age, and social status, but dementia caregivers reported slightly poorer self-rated health and higher signs of subclinical depression than non-caregivers (see Table 1).

As detailed below, no significant differences emerged between dementia caregivers and non-caregivers’ personal VoA. Regarding the role of sociodemographic and health factors, none of the predictors showed plausibility of explaining variance in felt age in both groups, and in AARC-Gains for dementia caregivers. Social status index was the only variable significantly and negatively associated with AARC-Gains among non-caregivers. The social status index and self-rated health were significantly, negatively associated with caregivers’ AARC-Losses scores, whereas older age and higher GDS scores were significantly associated with greater AARC-Losses among non-caregivers. CBI (but not NPI distress) emerged as an additional predictor, alongside sociodemographic and health indicators, associated only with greater AARC-Losses (but not felt age or AARC-Gains) among dementia caregivers. The path model revealed direct associations between AARC-Losses and CBI, social status index and self-rated health, as well as an indirect association between GDS and AARC-Losses through CBI.

### 3.1. Differences Between Caregivers and Non-Caregivers’ Personal VoA and the Role of Sociodemographic and Health Factors

Results from the ANOVAs showed no significant differences between groups for felt age or the AARC-Gains and AARC-Losses scores (see Table 1). To further ascertain any potential difference between dementia caregivers and non-caregivers’ personal VoA accounting for health measures, ANCOVAs were run with self-rated health and GDS as covariates, group (caregivers vs. non-caregivers) as the between-subject factor, and felt age, AARC-Gain, and AARC-Losses separately as dependent variables. The group effect was again not significant for any of the VoA measures considered (all F_s_ < 1). Significant effects of covariates emerged only for AARC-Losses (self-rated health, F_(1,160)_ = 9.208, *p* = 0.003, *η*^2^*_p_* = 0.054; GDS, F_(1,160)_ = 17.174, *p* < 0.001, *η*^2^*_p_* = 0.097).

For felt age, LM analyses revealed the null model (m0) as the most plausible for both dementia caregivers and non-caregivers (Table S1), suggesting that none of the predictors showed plausibility of explaining variance in felt age in both groups (see Table 3).

For AARC-Gains, the full model was the most plausible for dementia caregivers (m2; AICw = 0.368; R^2^ = 0.011; Table S1), but none of the considered variables was associated with AARC-Gains in this group (see Table 3).

The most plausible model for non-caregivers was the one with age and social status index (m1; AICw = 0.822; R^2^ = 0.107; Table S1). The social status index was the only variable associated with AARC-Gains, indicating that non-caregivers with higher social status reported lower awareness of age-related gains (Table 3).

For AARC-Losses, the full model (m2) was the most plausible among dementia caregivers (AICw = 0.969; R^2^ = 0.279; see Table S1). Social status index and self-rated health were significantly associated with caregivers’ AARC-Losses scores (see Table 3).

The full model (m2) was found to be the most plausible for non-caregivers as well (AICw = 1.000; R^2^ = 0.461; see Table S1). Age (albeit just at the set significant level) and GDS scores were significantly associated with AARC-Losses, indicating that older non-caregivers with higher subclinical symptoms of depression reported higher awareness of age-related losses (Table 3).

### 3.2. The Role of Caregiver Burden and Distress on Dementia Caregivers’ Personal VoA

The LM analyses considering dementia caregivers only confirmed that the null model (m0) and the models with sociodemographic characteristics and health outcomes (m2) remained the most plausible for felt age and AARC-Gains, respectively (see Table S2).

As for AARC-Losses, the model including also CBI scores (but not the one with NPI distress scores), alongside socio-demographic and health indicators, emerged as the most plausible (m3; AICw = 0.572, R^2^ = 0.380; Table S2). Social status, self-rated health, and CBI were significantly associated with AARC-Losses scores, indicating that caregivers with lower social status, poorer self-rated health, and greater burden reported higher awareness of age-related losses (see Table 4).

In the light of the model comparison results, a path analysis was performed, for exploratory purposes only, to clarify the pattern and strength of direct and indirect relationships among sociodemographic, health-related, and burden variables in explaining AARC-Losses.

The model estimated the direct effects of sociodemographic characteristics (age, social status index), self-rated health, and GDS on AARC-Losses scores, as well as their indirect effects through the direct effect of CBI on AARC-Losses scores.

**Figure 1:**
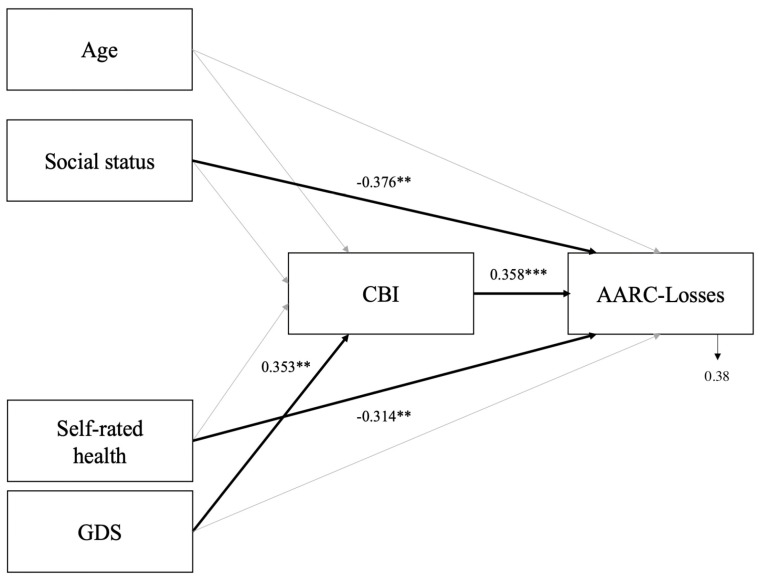
Results (standardized solutions) of the path model for AARC-Losses among dementia caregivers. ** *p* < 0.01; *** *p* = 0.001. Notes. Age: chronological age; AARC: Awareness of Age-Related Change; GDS: Geriatric Depression Scale; CBI: Caregiver Burden Inventory.

Results from the path model (R^2^ = 0.38; see Table S3 for a summary of the results) showed a direct association of CBI and AARC-Losses scores (B = 0.358, *p* < 0.01), as well as significant direct associations of the social status index (B = −0.376, *p* < 0.01) and self-rated health (B = −0.314, *p* < 0.01) on AARC-Losses scores (see Figure 1). A direct effect of GDS on CBI scores (B = 0.353, *p* < 0.01; see Figure 1) and an indirect effect of GDS mediated by CBI on AARC-Losses scores (B = 0.126, *p* = 0.023) also emerged (see Figure 1).

## 4. Discussion

This study aims to further elucidate individual differences between informal caregivers of PwD, exposed to the complex, stressful, ongoing task of caring for a person with this neurodegenerative disease, and non-caregiving peers in perceptions of their own aging, considering personal VoA facets. We specifically examined whether caring for a PwD and the experienced burden and distress derived from the occurrence of behavioral and psychological symptoms of dementia are related to dementia caregivers’ perceptions of their own aging in terms of felt age and their awareness of aging-related gains and losses (AARC-Gains and AARC-Losses).

In line with our expectations and previous evidence [9,10,11], dementia caregivers reported poorer self-rated health and more subclinical depressive symptoms than non-caregivers. Such a result confirms that caring for a PwD is related to a greater likelihood of experiencing negative physical and psychological sequelae as compared with peers who were never charged with this role [9,10,11].

Any differences between dementia caregivers and non-caregivers’ personal VoA, either in terms of felt age or AARC-Gains and AARC-Losses, emerged, even after controlling for health outcomes. This result aligns with previous scant evidence showing no clear differences between these two groups in terms of aging self-perceptions and experiences [20,25,26]. It might be that dementia is considered, independently of being a caregiver or not, as an inevitable consequence of becoming older, and such a belief might impact actual aging self-views and shape awareness of the aging self [32,52,53]. Thus, this might explain the null effect of caregiving per se on VoA, because such a role is seen as an integral and inescapable part of growing older.

Nonetheless, looking at the role of individual characteristics in personal VoA revealed some peculiarities. Sociodemographic and health factors contributed to explaining personal VoA to a different extent when felt age or AARC were considered.

None of the included variables, in fact, contributed to explaining felt age, neither in non-caregivers nor in dementia caregivers. Notwithstanding felt age being among the most widely used indicators of personal VoA, our results align with previous evidence suggesting that felt age is not always associated with or seems to show less consistent associations with health outcomes than more comprehensive, multidimensional measures of VoA [18]. A previous study found associations between dementia caregivers’ felt age and health conditions [24]. Nonetheless, felt age was measured with a Likert scale instead of using proportional discrepancy scores, and the sample comprised participants with depressive symptoms, which was not the case here; these discrepancies could explain such contrasting findings. Previous studies also found associations between felt age and socioeconomic status [54]. However, the latter, contrary to the present study, included individuals within a broader age range; felt age was computed as the discrepancy score between chronological age and felt age (not adjusting for chronological age as in this study), and socioeconomic status was conceived also in terms of household income and perceived financial well-being, as not shown here. These discrepancies could explain such contrasting results, and these aspects merit further investigation, especially among dementia caregivers, given the financial hardship that caring for a PwD could pose [4]. Although felt age is typically assessed with a single item question, as shown here, future studies among dementia caregivers should also attempt to better understand the interplay of sociodemographic and health-related outcomes with various dimensions of felt age (e.g., mental, physical, ideal felt age [17]).

As for AARC, different sociodemographic and health-related factors contributed to explaining awareness of age-related changes, to a different extent among dementia caregivers and non-caregiving peers when AARC-Gains or AARC-Losses were concerned.

Among non-caregivers, lower AARC-Gains were associated with higher social status, whereas higher AARC-Losses were associated with older age and more subclinical depressive symptoms. As for AARC-Gains, our findings mirror previous evidence showing that having a university education and being employed are associated with lower AARC-Gains [27,55]. Our results further suggest that, regardless of age and health status, engaging in a job, also in relation to the educational attainment acquired, might make individuals less sensitive and aware of positive age-related changes that could be experienced in various domains, as captured by the AARC-Gains subscale at least, such as taking better care of one’s own health, having time to enjoy life, pursuing hobbies or leisure activities, and cultivating meaningful social/family relationships [27,33]. As for AARC-Losses, our results, in line with previous evidence, further highlighted that self-perceptions of age-related losses are associated with being older and reporting poorer mental health outcomes. The greater salience of perceived losses, with increasing age linked to accumulating stressful life or health-related events [27,33,55], might thus account for a greater awareness of age-related losses.

With dementia caregivers, none of the sociodemographic and health-related variables were associated with AARC-Gains. Such an unexpected result could lie in the fact that other individual characteristics not examined here, such as coping strategies and personality dispositions, but also strengthened relationships and other positive aspects (e.g., personal growth, purpose, and fulfillment) known to be related to the caregiving experience [56], might more likely influence dementia caregivers’ perceptions and awareness of gains associated with increasing age [20,23,24]. It is worth mentioning that this is the first study, albeit cross-sectional, that thoroughly examined AARC among dementia caregivers, and these speculations warrant further investigation.

For AARC-Losses, associations with not only social status and health outcomes but also with caregiver burden—as measured by the CBI—emerged as significant. In particular, as exploratorily revealed by the path model, poorer social status, self-rated health, and a higher burden were directly associated with higher AARC-Losses scores, and an indirect cross-sectional effect of subclinical depressive symptoms exerted through the perceived burden of caregiving contributed to explaining dementia caregivers’ awareness of age-related losses. Such results further suggest that caregiving burden is particularly associated with psychological health (mood) issues among caregivers of PwD [10]. Note that in the present study, informal caregivers reported worse self-rated health and more subclinical depressive symptoms than non-caregiving peers. Moreover, they highlight that the lack of skills and opportunities, related to poorer social status (half of the dementia caregivers were retired, and 91% of these people did not have a university education), as well as the perceived lack of physical and psychological resources, related to poorer self-rated health, are associated with awareness of age-related losses, as usually found in studies examining AARC and extended here also to informal dementia caregivers [27,33,55]. These results also suggest that the more dementia caregivers struggle to cope with the care of their relative with dementia, experiencing burden derived from different task-related or emotional sources of care associated with subthreshold depression, the more they are likely to become aware of or raise their perceptions of their own age-related losses in different domains of functioning.

Interestingly, distress deriving from the behavioral and psychological symptoms of dementia, examined here with the NPI-Distress scale, did not emerge as a plausible correlate of the facets of personal VoA considered here among dementia caregivers. Such a result seems to conflict with our expectations and those of a previous study [24] showing an association between dementia caregivers’ felt age and perceived distress. Here again, the different ways in which felt age but also distress (examined with a generalized distress measure instead of using specific caregiving-related scales) were operationalized could explain such contrasting results. The NPI specifically assesses neuropsychiatric symptoms and psychopathology, referring to severity and frequency of PwD behavior and, more marginally, to emotional distress perceived by the caregiver. The CBI, in contrast, captures broader difficulties across different domains (spanning social, physical, and psychological constraints), reflecting the everyday life of the dementia caregiver. Thus, it could be more likely associated with caregivers’ self-perceptions of aging-related changes -losses- in such domains, as measured by AARC. This questionnaire, in fact, examines aging-related changes across different broad domains of functioning also related to everyday life. Noteworthy, however, is the fact that emotional burden is already one of the explored dimensions within the CBI [34,37]. The distress contribution to negative VoA cannot thus be excluded, as it is already included within the CBI. Future studies, using finer-graded objective burden and health-related measures along with other determinants of caregiver burden (e.g., relationship quality, caregiving duration, financial strain), should confirm and further our results.

It is also worth noting that PwD may exhibit different behavioral and psychological symptoms depending, among others, on dementia type, severity, onset, and duration [57,58,59,60,61], and such symptoms are perceived as more or less stressful by caregivers [57,58]. The salience of specific symptoms, as a function of care recipient dementia characteristics, might more likely shape caregivers’ personal VoA. Although we attempted to collect the most up-to-date information about care-recipient profiles, they were caregiver-reported and lacked dementia onset and duration. Moreover, the sample size and the exploratory nature of this study did not allow further investigation of this speculation, which should be addressed in future research.

It should also be acknowledged that the involvement of a convenience sample of Italian middle-aged, predominantly female adults and older adults and, in the case of dementia caregivers, supported by a network of healthcare services, may affect the generalizability of our results to a broader population or to more heterogeneous samples of informal dementia caregivers. Future studies should also try to analyze the role of societal/cultural differences as well as of gender differences, and particularly the needs and experiences of men caring for PwD [62], not considered here because of the large number of female caregivers. Finally, research adopting a longitudinal approach including other nuances of VoA (e.g., [non]essentialist beliefs about aging, attitudes toward aging [12]) is also warranted to confirm and elucidate the associations between caregivers’ characteristics, caregiving-related sequelae and VoA among dementia caregivers. 

Nonetheless, all said, our study is among the first highlighting a link between caring for a PwD and informal dementia caregivers’ views of their own aging self, thereby offering insight to deepen this issue further.

## 5. Conclusions

Caring for a PwD, as also found here, increases the likelihood of experiencing negative physical and psychological sequelae as compared with non-caregiving peers. Though VoA did not differ between caregivers and non-caregiving peers, our results show a specific interplay between the mood and burden associated with caring for a person with dementia and caregivers’ personal VoA, particularly regarding awareness of age-related losses. Such findings offer valuable insight for further research on the links between dementia caregiving, related strains, and VoA.

From an applied perspective, integrating the assessment of VoA into the evaluation of burden and health-related consequences of caregiving in clinical practice would provide a more comprehensive understanding of dementia caregivers’ experiences and perceptions of their own general aging process, especially biased ones related to awareness of age-related changes in terms of losses and not gains.

Notably, growing evidence advocates that individuals’ VoA are not immutable, yet amenable to modification and optimization, thereby representing psychological resources that can be activated to foster healthy and successful aging [63]. Consequently, public policies and interventions that promote more positive attitudes and self-perceptions of aging, and eradicate negative stereotypes commonly used to designate older people, especially among dementia caregivers, are crucial. Alongside strategies to manage dementia and sources of care burden, caregiving interventions would benefit from the inclusion of psychoeducational components that provide accurate information regarding typical/atypical aging experiences, dispel negative age stereotypes, strengthen growth/positive mental representation and experiences of one’s own aging process, and prompt strategies to control and cope with aging, thereby helping caregivers continue aging well while caring for a relative with dementia.

## Figures and Tables

**Table 1:** Descriptive statistics for the sociodemographic characteristics and measures of interest by group, differences between groups, and care-recipients’ characteristics.

	Caregivers (N = 70)		Non-Caregivers (N = 94)		Groups Differences		
	*M*	*SD*	*M*	*SD*	*F*_(1,162)_	*p*	η^2^_p_
Chronological age (years)	62.14	10.34	62.21	10.09	<1		
Hollingshead Social Status index	22.26	17.47	24.18	18.66	<1		
*Education (years)*	11.39	3.58	12.20	3.59			
*Unemployed or retired, n (%)*	36 (51.4%)		50 (53.2%)				
Self-rated health	3.60	0.67	3.82	0.56	5.35	0.022	0.032
GDS	2.41	1.52	1.68	1.52	9.36	0.003	0.055
Felt age	−0.13	0.14	−0.12	0.12	<1		
AARC-Gains	82.86	17.02	85.15	13.79	<1		
AARC-Losses	57.27	14.68	53.40	13.81	2.99	0.086	0.018
NPI-Distress	13.99	11.12	-	-			
CBI	32.10	14.54	-	-			
Cared for, n (%)			-				
*Spouse*	21 (30.00%)						
*Mother/father (in-law)*	49 (64.3%)						
CR dementia type, n (%)			-				
*Alzheimer’s*	26 (37.1%)						
*Vascular*	10 (14.3%)						
*Mixed*	11 (15.3%)						
*Other*	23 (32.9%)						
CR dementia severity, n (%)			-				
*Mild (MMSE score: 27–20)*	23 (32.9%)						
*Moderate (MMSE score: 19–13)*	29 (41.4%)						
*Severe (MMSE score: 12–0)*	18 (25.7%)						

Notes. GDS: Geriatric Depression Scale; AARC: Awareness of Age-Related Change; NPI: Neuropsychiatric Inventory; CBI: Caregiver Burden Inventory; CR: care recipient; MMSE: Mini-Mental State Examination.

**Table 2:** Summary of the models tested to investigate the relationships between sociodemographic factors (chronological age and social status), self-rated health, depression, and dementia caregiver’s burden and distress with felt age, AARC-Gains, and AARC-Losses.

Model	Predictors
m0	′y ~ intercept
m1	′y ~ intercept + chronological age + social status index
m2	′y ~ intercept + chronological age + social status index + self-rated health + GDS
m3	′y ~ intercept + chronological age + social status index + self-rated health + GDS + CBI
m4	′y ~ intercept + chronological age + social status index + self-rated health + GDS + NPI-Distress scores
m5	′y ~ intercept + chronological age + social status index + self-rated health + GDS + CBI + NPI-Distress

Notes. GDS: Geriatric Depression Scale; NPI: Neuropsychiatric Inventory; CBI: Caregiver Burden Inventory.

**Table 3:** Results of the best models for felt age and AARC-Gains and Losses scores for caregivers and non-caregivers.

		Predictors	B	CI		*p*
Dementia caregivers	Felt age	Intercept	−0.130	−0.164	−0.096	<0.001
	AARC-Gains	Intercept	76.267	23.267	129.267	0.005
		Age	−0.111	−0.633	0.410	0.671
		Social status index	−0.216	−0.509	0.075	0.143
		GDS	−0.782	−3.625	2.061	0.584
		Self-rated health	5.614	−1.263	12.493	0.107
	AARC-Losses	Intercept	90.324	49.148	131.499	<0.001
		Age	−0.009	−0.415	0.395	0.961
		Social status index	−0.310	−0.537	−0.083	0.008
		GDS	0.692	−1.516	2.901	0.533
		Self-rated health	−7.555	−12.899	−2.211	0.006
Non-caregivers	Felt age	Intercept	−0.122	−0.146	−0.098	<0.001
	AARC-Gains	Intercept	95.672	70.486	120.858	<0.001
		Age	−0.066	−0.417	0.284	0.706
		Social status index	−0.263	−0.453	−0.073	0.007
	AARC-Losses	Intercept	35.091	11.094	59.088	0.005
		Age	0.352	0.063	0.642	0.017
		Social status index	−0.107	−0.266	0.051	0.184
		GDS	4.177	2.558	5.795	<0.001
		Self-rated health	−2.109	−6.559	2.341	0.348

Notes. AARC: Awareness of Age-Related Change; age: chronological age; GDS: Geriatric Depression Scale.

**Table 4:** Results of the best models for felt age and AARC-Losses when adding burden as predictors for dementia caregivers only.

Measure of interest	Predictors	B	CI		*p*
AARC-Losses	Intercept	84.637	45.972	123.302	<0.001
	Age	−0.096	−0.479	0.286	0.614
	Social status index	−0.316	−0.528	−0.103	0.004
	GDS	−0.529	−2.729	1.670	0.632
	Self-rated health	−6.836	−11.853	−1.819	0.008
	CBI	0.360	0.137	0.584	0.002

Notes. Age: chronological age; GDS: Geriatric Depression Scale; CBI: Caregiver Burden Inventory.

## Data Availability

The data are not readily available, due to the confidential nature of (part of) the dataset we collected.

## Supplementary Materials

The following supporting information can be downloaded at: https://www.mdpi.com/article/10.3390/healthcare13222884/s1, Table S1. Model comparison results for non-caregivers and dementia caregivers; Table S2. Model comparison results for dementia caregivers only when adding distress and burden as predictors; Table S3. Summary of the results of the direct and indirect effects in the path model for AARC-Losses among dementia caregivers.
