# Supplementary material for: Personal Views of Aging Among Informal Caregivers of People with Dementia and Non-Caregivers: Gauging the Role of Individual Characteristics and Caregiving-Related Burden
Source: Healthcare (Basel). 2025 Nov 12;13(22):2884. doi: 10.3390/healthcare13222884 (PMC12652656; doi:10.3390/healthcare13222884)
Supplement: Supplementary file 1 [file healthcare-13-02884-s001.zip › healthcare-3935801-supplementary.pdf]

Supplemental Materials

Personal views of aging among informal caregivers of people with dementia and non-caregivers: gauging the role of individual characteristics and caregiving-related burden.

Table S1. *Model comparison results for non-caregivers and dementia caregivers. The most parsimonious, best-fit model, i.e., with the lowest AIC and highest weights, selected for each measure of interest (felt age, AARC-Gains and AARC-Losses) appears in bold.*

Measure of interest	Model	Predictors	AIC	AICw	R^2	adj R^2
**Non-caregivers**						
Felt age	**m0**	**Intercept**	**-134.23**	**0.569**	**0**	**0**
	m1	Intercept+ss	-132.83	0.282	0.006	-0.004
	m2	Intercept+ss+GDS+self-rated health	-131.56	0.149	0.034	0.002
AARC-Gains	m0	Intercept	763.08	0.029	0	0
	**m1**	**Intercept+age+ss**	**756.41**	**0.822**	**0.107**	**0.087**
	m2	Intercept+age+ss+GDS+self-rated health	759.83	0.148	0.112	0.072
AARC-Losses	m0	Intercept	763.45	0	0	0
	m1	Intercept+age+ss	743.73	0	0.223	0.205
	**m2**	**Intercept+age+ss+GDS+self-rated health**	**713.35**	**1**	**0.461**	**0.436**
**Dementia caregivers**						
Felt age	**m0**	**Intercept**	**-70.73**	**0.560**	**0**	**0**
	m1	Intercept+ss	-69.87	0.364	0.016	0.001
	m2	Intercept+ss+GDS+self-rated health	-66.73	0.076	0.028	-0.015
AARC-Gains	m0	Intercept	598.47	0.313	0	0
	m1	Intercept+age+ss	598.43	0.319	0.056	0.027
	**m2**	**Intercept+age+ss+GDS+self-rated health**	**598.15**	**0.368**	**0.112**	**0.057**
AARC-Losses	m0	Intercept	577.80	0.001	0	0
	m1	Intercept+age+ss	569.73	0.030	0.158	0.133
	**m2**	**Intercept+age+ss+GDS+self-rated health**	**562.81**	**0.969**	**0.279**	**0.235**

Notes. AARC: Awareness of Age-Related Change; age: chronological age; ss: social status index; GDS: Geriatric Depression Scale.

Table S2. *Model comparison results for dementia caregivers only when adding distress and burden scores as predictors. The most parsimonious, best-fit model, i.e., with the lowest AIC and highest weights, selected for each measure of interest (felt age, AARC-Gains and AARC-Losses) appears in bold.*

Measure of interest	Model	Predictors	AIC	AICw	R^2	adj R^2
Felt age	**m0**	**Intercept**	**-70.73**	**0.289**	**0**	**0**
	m1	Intercept+ss	-69.87	0.188	0.016	0.001
	m2	Intercept+ss+GDS+self-rated health	-66.73	0.039	0.028	-0.015
	m3	Intercept+ss+GDS+self-rated health+CBI	-68.96	0.120	0.085	0.028
	m4	Intercept+ss+GDS+self-rated health+NPI-Distress	-70.12	0.213	0.100	0.044
	m5	Intercept+ss+GDS+self-rated health+CBI+NPI-Distress	-69.42	0.151	0.116	0.047
AARC-Gains	m0	Intercept	598.47	0.233	0	0
	m1	Intercept+age+ss	598.43	0.238	0.056	0.027
	**m2**	**Intercept+age+ss+GDS+self-rated health**	**598.15**	**0.274**	**0.112**	**0.057**
	m3	Intercept+age+ss+GDS+self-rated health+CBI	600.00	0.109	0.114	0.044
	m4	Intercept+age+ss+GDS+self-rated health+NPI-Distress	600.11	0.103	0.112	0.043
	m5	Intercept+age+ss+GDS+self-rated health+CBI+NPI-Distress	601.81	0.044	0.116	0.032
AARC-Losses	m0	Intercept	577.80	0	0	0
	m1	Intercept+age+ss	569.73	0	0.158	0.133
	m2	Intercept+age+ss+GDS+self-rated health	562.81	0.008	0.279	0.235
	**m3**	**Intercept+age+ss+GDS+self-rated health+CBI**	**554.29**	**0.572**	**0.380**	**0.332**
	m4	Intercept+age+ss+GDS+self-rated health+NPI-Distress	559.30	0.047	0.334	0.282
	m5	Intercept+age+ss+GDS+self-rated health+CBI+NPI-Distress	555.14	0.373	0.390	0.332

Notes. AARC: Awareness of Age-Related Change; age: chronological age; ss: social status index; GDS: Geriatric Depression Scale; CBI: Caregiver Burden Inventory; NPI: NeuroPsychiatric Inventory.

Table S3. *Summary of the results (standardized solutions) for the direct and indirect effects tested in the path model for AARC-Losses among dementia caregivers.*

**Direct effects**		B	*z*	*p*
AARC-Losses				
	**CBI**	**0.358**	**3.369**	**0.001**
	Age	-0.068	-0.529	0.597
	**ss**	**-0.376**	**-3.111**	**0.002**
	**Self-rated health**	**-0.314**	**-2.847**	**0.004**
	GDS	-0.055	-0.503	0.615
CBI				
	Age	0.172	1.193	0.233
	ss	0.018	0.132	0.895
	Self-rated health	-0.092	-0.746	0.455
	**GDS**	**0.353**	**3.070**	**0.002**
**Indirect effects**				
AARC-Losses				
	Age	0.061	1.124	0.261
	ss	0.006	0.132	0.895
	Self-rated health	-0.033	-0.729	0.466
	**GDS**	**0.126**	**2.269**	**0.023**

Notes. AARC: Awareness of Age-Related Change; age: chronological age; ss: social status index; GDS: Geriatric Depression Scale; CBI: Caregiver Burden Inventory.
